# Exploring Combined Effect of Abiotic (Soil Moisture) and Biotic (*Sclerotium rolfsii* Sacc.) Stress on Collar Rot Development in Chickpea

**DOI:** 10.3389/fpls.2018.01154

**Published:** 2018-08-15

**Authors:** Avijit Tarafdar, T. Swaroopa Rani, U. S. Sharath Chandran, Raju Ghosh, Devashish R. Chobe, Mamta Sharma

**Affiliations:** Legumes Pathology, Integrated Crop Management, International Crops Research Institute for the Semi-Arid Tropics, Patancheru, India

**Keywords:** chickpea, collar rot, gene expression, *Sclerotium rolfsii*, soil moisture

## Abstract

Plants being sessile are under constant threat of multiple abiotic and biotic stresses within its natural habitat. A combined stress involving an abiotic and a biotic factor reportedly increases susceptibility of the plants to pathogens. The emerging threat, collar rot disease of chickpea (caused by *Sclerotium rolfsii* Sacc.) is reported to be influenced by soil moisture condition (SMC). Hence, we studied the influence of differential SMC *viz*. upper optimum (100%), optimum (80%), lower optimum (60%), and limiting (40%) soil moisture conditions on colonization and collar rot development over the course of infection in two chickpea cultivars, Annigeri (susceptible to collar rot) and ICCV 05530 (moderately resistant to collar rot). Disease incidence was found to be directly proportional to increase in soil moisture (R^2^ = 0.794). Maximum incidence was observed at 80% SMC, followed by 100 and 60% SMC. Expression of genes (qPCR analysis) associated with host cell wall binding (lectin) and degradation *viz*. endopolygalacturonase-2, endoglucosidase, and cellobiohydrolase during collar rot development in chickpea were relatively less at limiting soil moisture condition (40%) as compared to optimum soil moisture condition (80%). As compared to individual stress, the expression of defense response genes in chickpea seedlings were highly up-regulated in seedlings challenged with combined stress. Our qPCR results indicated that the expression of defense-related genes in chickpea during interaction with *S. rolfsii* at low SMC was primarily responsible for delayed disease reaction. Involvement of moisture and biotic stress-related genes in combined stress showed a tailored defense mechanism.

## Introduction

Chickpea (*Cicer arietinum* L.) is one of the most important and essential legumes crops for semi-arid tropical area. India is the largest producer of chickpea, accounts 70.9 % of the world cultivated area and produces 67.1 % of the total world production (FAOSTAT, 2013). The vulnerability of chickpea to biotic and abiotic stresses is major constraint for reduced yields. The on-going changes in climatic conditions such as increase in CO_2_ emissions, unpredicted rainfall patterns, temperature rise, decrease/increase in relative humidity, and low soil moisture stress (Zhao and Running, 2010) are likely to influence the plant diseases establishment, its distribution and epidemiology (Graham and Vance, 2003). Consequently, the evidences suggest major shift in the chickpea soil-borne diseases like dry root rot (*Rhizoctonia bataticola*), Fusarium wilt (*Fusarium oxysporum* f. sp. *ciceris*), collar rot (*Sclerotium rolfsii*), wet root rot (*Rhizoctonia solani*) and black root rot (*Fusarium solani*) in the semi-arid tropic (SAT) regions.

As we know, plants in fields are always exposed to multiple biotic and abiotic stresses where plants exhibit certain unique and convergent physiological and molecular responses that interact and impact each other to withstand the combined effect of these stresses (Choi et al., 2013; Padaria et al., 2015; Pandey et al., 2015). These combined stress interactions in plants may either have direct or indirect effect on pathogens through other community interactions (Sharma and Ghosh, 2017) leading to either positive or negative effects on plant responses (Ramegowda et al., 2013). In a combined stress scenario, drought can affect the pathogen infection either positively or negatively (Graham and Vance, 2003). Previous reports have shown that susceptibility of plants to bacterial pathogens tend to increase under low soil moisture stress (Mohr and Cahill, 2003; Choi et al., 2013). In chickpea, low soil moisture stress significantly increased the incidence of dry root rot caused by *Rhizoctonia bataticola* (Sharma and Pande, 2013). Conversely, reports also indicated that low soil moisture stress improves the defense response of plants against pathogens (Ramegowda et al., 2013; Hatmi et al., 2015; Sinha et al., 2016). On the other hand, it has also been found that not only low but high soil moisture condition also favors some diseases (Blaker and MacDonald, 1981; Ferraz et al., 1999). Therefore, it is crucial to understand the effect of combined stress and the respective defensive strategies adopted by the plants to overcome the synchronous onslaught of low soil moisture stress and pathogen. The molecular responses of different crops against several pathogen infection and combined low soil moisture stress have been reported (Choi et al., 2013; Ramegowda et al., 2013; Hatmi et al., 2015). However, so far no attempt has been made to understand the molecular responses of chickpea to combined soil moisture stress with soil borne fungal infections. Among soil borne diseases in chickpea, dry root rot and collar rot are predisposed by low and high soil moisture respectively. There are some reports of effects of soil moisture and temperature on dry root rot of chickpea but minimal work has been done with respect to collar rot.

Collar rot is an emerging soil-borne disease of chickpea that may incite 55–95 % mortality of chickpea seedlings under favorable environmental conditions like heavy rainfall and high soil temperature (25–30°C) (Sharma and Ghosh, 2017). Moreover, collar rot management is quite challenging owing to the pathogens wide host range including at least 500 species coming under 100 families commonly in legumes, crucifers, and cucurbits (Aycock, 1966). *Sclerotium rolfsii* survives in the form of mycelium in the infected tissues and plant debris and as sclerotial structures in the soil or in association with plant debris and usually attacks the collar region of plants. Because of high competitive saprophytic survival ability, in recent years, *S. rolfsii* is becoming more prevalent in agricultural areas where sudden rainfall increases soil moisture for longer periods combined with warm temperatures. With the availability of such a large range of natural hosts, *S. rolfsii* could even survive in dry climatic regions and continue to persist in the soil for prolonged periods even after several crop rotations. Lack of sufficient information regarding the factors affecting collar rot development have made its control quite difficult.

The present study was therefore aimed to understand the influence of differential soil moisture stress on the severities of *S. rolfsii* infection in chickpea. Attempts have been made to study the differences in the net impact of combined stress compared to the respective individual stresses at molecular level including differential gene expression. To the best of our knowledge this is the first report showing the molecular responses of chickpea during individual and combined biotic (*S. rolfsii*) and abiotic (soil moisture) stress interactions.

## Materials and methods

### Plant material and growth conditions

For the present investigation, we have considered two chickpea cultivars Annigeri and ICCV 05530 based on their resistance and susceptibility reaction to collar rot (*S. rolfsii*). Annigeri is highly susceptible and ICCV 05530 is moderately resistant to collar rot. The latter is also found to be resistant/moderatly resistant to other soil borne diseases-*Fusarium* wilt and dry root rot in chickpea (Tarafdar et al., 2017). Apparently healthy seeds of both cultivars were surface sterilized with 2% sodium hypochloride (NaOCl) for 2 min followed by two times washing with sterile deionized water. Seven seeds per pot were sown in 15 cm plastic pots containing sterilized vertisol and sand (3:1) and kept in a growth chamber with controlled environment having a diurnal cycle of 14 h light/10 h dark with 28 ± 1°C and 50–60% relative humidity.

### Fungal isolate and inoculum preparation

A pathogenic isolate of *S. rolfsii* viz. Sr 1, isolated from infected chickpea plant from experimental field of ICRISAT, Patancheru, Telangana, India was used in the experiments (Tarafdar et al., 2017). For mass multiplication of *S. rolfsii* inoculum, 100 g seeds of sorghum were soaked in water for 2 days and 2 ml of 2% sucrose solution was added prior to autoclave. Four plugs of actively growing 4-day-old *S. rolfsii* culture on PDA were inoculated into the autoclaved sorghum grains and incubated at 28 ± 1°C for 7 days.

### Soil moisture stress imposition

Effect of different soil moisture conditions on the development of collar rot disease in chickpea was studied. Seven-day-old chickpea seedlings grown in pre-weighted pots were divided into four different sets with three replications and pots were maintained in four different soil moisture conditions(SMC), i.e., upper optimum soil moisture (100%), optimum soil moisture (80%), lower optimum soil moisture (60%), and limiting soil moisture (40%) conditions (Table 1). The SMC was maintained by withholding the water supply and determined by means of gravimetric method on oven dry basis as described earlier (Sharma and Pande, 2013). To obtain all the four moisture condition at same time, water withdrawal for each moisture level was done in batches. For imposing 40% soil moisture stress in chickpea seedlings, water withdrawal in pots was started 5 days prior inoculation, including 5 days of acclimatization. Similarly, for 60% SMC, water withdrawal was started 4 days before inoculation and for 80% SMC 2 days before inoculation.

**Table 1:** Details of experimental set up used to study *Sclerotium rolfsii* and chickpea interaction and summary of the observation.

**Parameter**			**Remarks**
**Soil moisture conditions (%)**	**Description**	**Pathogen**	
40	Limiting soil moisture	Inoculated	i.*S. rolfsii* colonization initiation at 24 hpi. Low pathogen growth even at 48 hpi due to low soil moisture condition. ii.Physiological wilting occurred due to purely abiotic stress in plant. iii.No plant mortality. iv.Not considered for gene expression study.
		Non-inoculated	i.Purely abiotic stress for plant ii.Considered for plant defence gene expression study w.r.t abiotic stress (48 h)^†^
60	Lower optimum soil moisture	Inoculated	i.Delayed *S. rolfsii* colonization initiation at 24 hpi due to lower optimum soil moisture condition and moderate disease incidence at 48 hpi. ii.Considered as combined (both biotic and abiotic) stress for plant and abiotic stress for pathogen growth. iii.Considered for gene expression studies for plant defense-related genes w.r.t combined stress and pathogenicity-causing genes w.r.t abiotic stress.
		Non-inoculated	i.Lower optimum soil moisture condition for plant. ii.Not considered for gene expression study.
80	Optimum soil moisture	Inoculated	i.Early initiation of *S. rolfsii* colonization at 12 hpi. Progressive colonization and high disease incidence due to optimum growth condition for both chickpea and *S. rolfsii*. ii.Purely biotic stress for plant growth iii.Optimum condition for studying host-pathogen interaction w.r.t plant defence genes and its expression. iv.Considered for study biotic stress.
		Non-inoculated	i.Optimum soil moisture for plant. ii.Considered as experimental control condition for plant growth. iii.Taken as control to normalize the gene expression profiles of plant defence-related genes.
100	Upper optimum soil moisture	Inoculated	i.Delayed *S. rolfsii* colonization initiation at 24 hpi due to high moisture condition, but fast growth of pathogen after the initial colonization led to disease incidence at par with optimum soil moisture condition at 48 hpi. ii.Upper optimum soil moisture for plant growth as well as pathogen growth. iii.Not considered for gene expression study.
		Non-inoculated	i.Upper optimum soil moisture for plant growth. ii.Not considered for gene expression study.

† *From the moisture stress point of view, the sampling time of 6–24 h is very short for inducing any significant abiotic stress in plants for gene expression studies, hence we have consider only 48 h sample in gene expression study w.r.t abiotic stress*.

### Combined soil moisture and pathogen stress imposition

Twelve days old chickpea seedlings maintained at different SMC were inoculated with *S. rolfsii* to understand the impact of SMC on development of collar rot disease in chickpea. *S. rolfsii*-infested sorghum grains were placed near the collar region of chickpea seedlings. Plants inoculated with sterile sorghum seeds served as mock. Different SMC were maintained in pots (described in above section) by regularly weighing each pot for the moisture deficit and replacing it by adding de-ionized water till the end of an experiment. The experiment was conducted in completely randomized design (CRD) and the disease incidence was recorded every day till the mortality of plants. Disease incidence was calculated by following formula: Disease incidence (%) = Total number of infected plants/total number of plants × 100. Correlation between disease incidence and SMC was observed by establishing the regression model, calculated at 1% level of significance at different moisture conditions.

### Quantification of *S. rolfsii* colonization

For quantification of *S. rolfsii* colonization, the samples (the shoot region up to 1 cm immediately adjacent to the infected collar region) were harvested from cv. Annigeri at 6, 12, 24, and 48 h post-inoculation (hpi), quick freezed in liquid N_2_, and preserved at −80°C for downstream experiments.

Genomic DNA was extracted from infected plant samples (cv. Annigeri) grown in different SMC and SR 1 isolate using PureLink Plant Total DNA Purification kit (Invitrogen, USA) as per the manufacturer's protocol. 100 mg of the harvested tissue was finely ground using liquid N_2_ and resuspended in 250 μL Resuspension buffer supplied in the kit. The resuspended tissue was vigorously vortexed until the samples homogenized completely. To lyse the tissues and avoid the RNA contamination, about 15 μL each of 20 % SDS and RNase (20 mg/ mL) was added to the tissue resuspension mixture and incubated for15 min at 55°C. The total gDNA from the sample was eluted by adding 50 μL of Elution buffer and stored at −20°C (Ghosh et al., 2017). Purified DNA was checked on 0.8% agarose gel and the extracted DNA was stored at −20°C for further use.

The absolute quantification of *S. rolfsii* DNA was measured through qPCR. The primers, qSR_5.8S (F), and qSR_5.8S (R) were designed from conserved region of the 5.8S sequences (Soeta et al., 2009) using IDT Primer Quest software (eu.idtdna.com/Primerquest/Home/Index;) (Table S1). DNA isolated from infected plants was used as template in qPCR to quantify fungal colonization. qPCR was carried out in Eppendorf Realplex Master Cycler (Eppendorf, Hamburg, Germany) using 10 μL reaction mixture consisting of 5 μL 2X KAPA SYBR Green PCR master mix (KAPA Biosystems, USA), 500 nM of each primer (qSR_F and qSR_R) and 1 ng of each template DNA. The PCR thermal cycling conditions were as follows: 95°C for 3 min followed by 40 cycles of 95°C for 10 s (denaturation), and 62°C for 30 s (annealing and extension) at which the fluorescence was measured and subsequently a melting curve was constructed by measuring continuous fluorescence at 60–95°C with increase of 0.5°C per second.

The standard curve (a plot of the Ct value vs. log DNA concentration) was prepared by following the protocol of Sharma et al. (2015) for quantifying the fungal DNA. The pure DNA of *S. rolfsii* was 10-fold serially diluted ranging from 10 ng to 0.01 pg and threshold cycle (Ct) for amplification of each diluted DNA was determined in qPCR under the same reaction conditions described above. Each sample amplification was conducted in triplicates in every experiment. The statistical significance of the difference in pathogen relative quantification at different soil moisture conditions was calculated by two-way ANOVA using Genstat 18.

### Real-time quantitative analysis of gene expression

For validation of gene(s) expression, total plant RNA was isolated from harvested plant samples (cv. Annigeri and ICCV 5530) at different hpi (6, 12, 24, and 48 hpi using GSure Tissue RNA kit (GCC Biotech, Kolakata, India) by following manufacturer's instruction. About 25–30 mg of tissue sample was ground in liquid nitrogen (N_2)_ and suspended 250 μL of Buffer GRT1. The suspension was incubated at 70°C for 15 min and vortexed it every 2 min interval. In final step total RNA was eluted in 50 μL of nuclease-free water. Purified RNA was quantified in Nanodrop spectrophotometer and evaluated on 1% agarose gel. The RNA was stored at −20°C for further downstream process.

A total of 1 μg of RNA in 20 μl reaction mixture was used for cDNA synthesis following the manufacture's protocol of Super Script III cDNA synthesis kit (Thermo Fisher, USA) following manufacturer's instruction. The expression profiling of genes causing pathogenicity in *S. rolfsii* and defense-related genes in chickpea were assessed. The 5.8s gene of *S. rolfsii* and actin of chickpea were used as endogenous reference genes for normalizing the gene expression (James et al., 2015). The primers used in expression analysis of qPCR assays are shown in Table S1. All the qPCR was carried out in 10 μL reaction mixtures as described above. Expression profile of each gene was determined by averaging of Ct value of three technical replicates from three biological replications. The primer specificity was confirmed using melting curve analysis. Relative expression of the genes was calculated by the 2^−DDCT^ method using Ct value (Livak and Schmittgenm, 2001). The statistical significance of the difference in relative expression at different soil moisture conditions in different chickpea cultivars was calculated by three-way ANOVA using Genstat 18.

## Results

### Assessment of fungal pathogenicity and combined stress imposition

**Figure 1:**
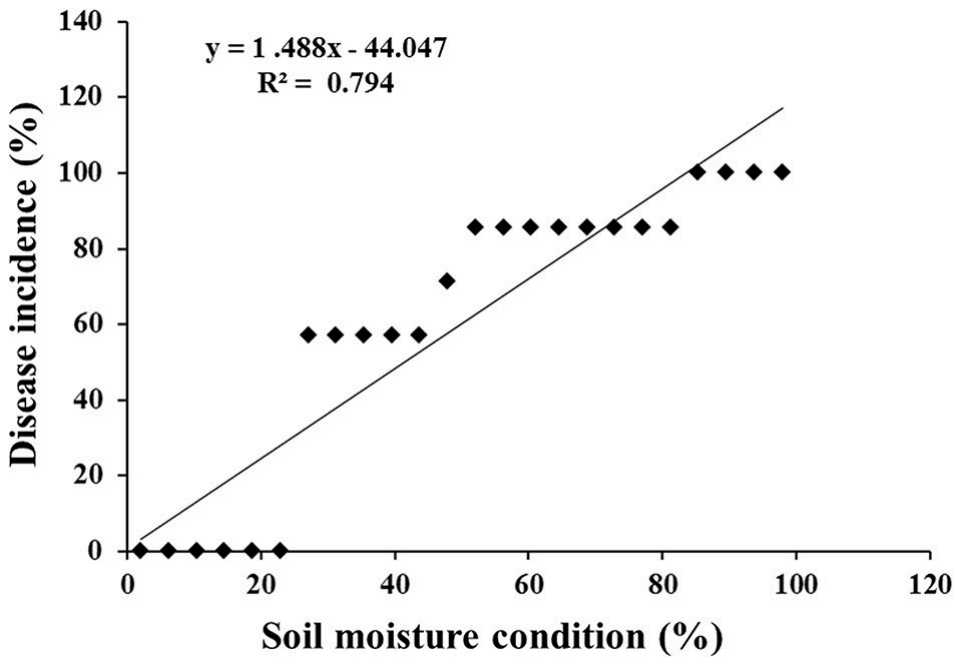
Scatter plot with corresponding regression line and regression equation for the relationship between the dependent variable disease incidence (%) and independent variable soil moisture condition (%).

At 48 hpi, the infected chickpea plants exposed to lower optimum (60%), optimum (80%), and upper optimum (100%) conditions revealed cellular degradation with tissue maceration and rotting of the collar region ultimately resulting in death of the plants. There was significant delay in the disease progression in ICCV 05530 as compared to Annigeri (*p* < 0.05; Table S2). At 48 hpi, the disease incidence was 9.5 and 4.8% in ICCV 05530 under optimum and upper optimum SMC, whereas, in Annigeri, the incidence was 76.2 and 71.4% respectively. Similar trend of disease development was observed up to 96 hpi in both the cultivars. However, disease incidence was at par in both the cultivars at 144 hpi. Collar rot incidence was lower during the combined application of low soil moisture and pathogen. There was a positive relationship (R^2^ = 0.794) between soil moisture (independent variable) and disease incidence (dependent variable) where, disease incidence (y) = (– 44.047) + 1.488x. However, it was observed that once disease appeared, the disease progression was faster in upper optimum soil moisture condition as compared to optimum soil moisture condition and no significant effect of soil moisture on collar rot incidence was observed after a certain period of inoculation (48 and 72 hpi; Figure 1). Under limiting soil moisture (40%) condition, no disease symptoms were observed in both chickpea cultivars even after 8 days of inoculation. Further, upon prolonged stress conditions (40% SMC), both inoculated and un-inoculated plants showed physiological wilting, indicating that longer exposure to insufficient moisture condition inhibited the normal growth of plants. No symptoms were recorded in un-inoculated control plants. On the other hand, it was observed that chickpea grown in upper optimum soil moisture condition had significantly taller stature with higher root and shoot biomass as compared to those from lower moisture condition.

### Fungal biomass vs. time-course of infection progression

**Figure 2:**
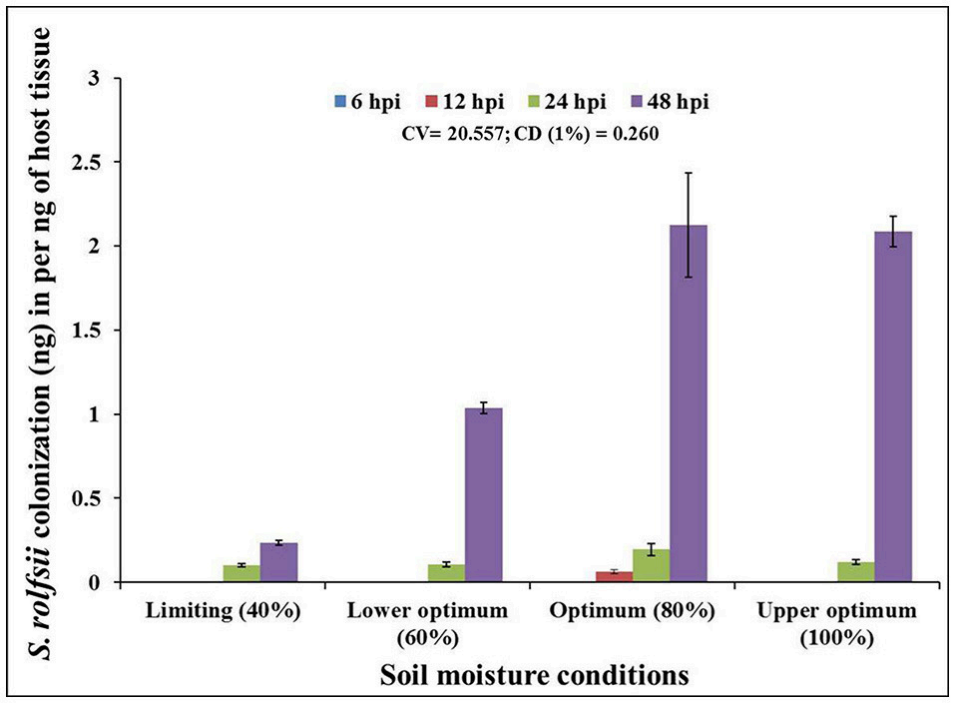
Chronological colonization profile of *S. rolfsii* in shoot tissues of inoculated chickpea (cv. Annigeri) grown in different soil moisture conditions. Absolute quantification of fungal DNA was determined in real-time PCR assay using sequences of 5.8S rDNA. Error bar represents the standard error of three biological replications at 95% confidence interval. The statistical significance between *S. rolfsii* growth and different soil moisture conditions was calculated by factorial ANOVA.

In order to determine the infection and colonization pattern of *S. rolfsii* in chickpea plants as affected by different soil moisture conditions, the fungal biomass within infected plant tissue (shoot) of cv. Annigeri was quantified using real-time qPCR assay. Absolute quantity of *S. rolfsii* DNA in infected chickpea plants was measured by assaying the *S. rolfsii* 5.8s gene using standard curve generated by 10-fold over 7-log range from 100 to 1 × 10^−3^ ng/ μL serially diluted *S. rolfsii* DNA (Figure S1). The slope of linear regression curve and correlation coefficient (R^2^) were −3.278 and 0.998 respectively, demonstrating the PCR efficiency of 101.87%. The calculated value for the limit of detection (LOD) at 95% level 2.9, which indicate heterogeneity in biological replication. For normalization of gDNA, the coefficient of variation (CV) was determined using gene copy number analysis. The CV of gene copy number was 33.13. The quantity of fungal DNA was recorded to exponentially increase in the host tissues over time, at all the soil moisture conditions except at limiting soil moisture condition At all the soil moisture conditions, no detectable amount of *S. rolfsii* biomass was measured within the host tissue during early stages of infection (at 6 hpi). However, the early initiation of *S. rolfsii* colonization was noted in 12 hpi at optimum soil moisture condition. Conversely, at 48 hpi, highest colonization of *S. rolfsii* from shoot tissue was recorded at all the soil moisture conditions. After 24 hpi the colonization of the fungus at lower optimum soil moisture condition was comparatively low with respect to succeeding higher moisture conditions (Figure 2). This correlated with the corresponding disease incidence at the respective time point. The highest fungal biomass of nearly 9-fold increase from limiting soil moisture condition and up to 2-fold increase from lower optimum soil moisture condition was recorded at optimum soil moisture condition closely followed by upper optimum soil moisture condition at 48 hpi. Even though there was successful infection at limiting soil moisture condition at 48 hpi, the colonization was not sufficient enough to lead to plant mortality (Figure 2).

### Expression analysis of selected pathogenicity-causing genes of *S. rolfsii* in chickpea

**Figure 3:**
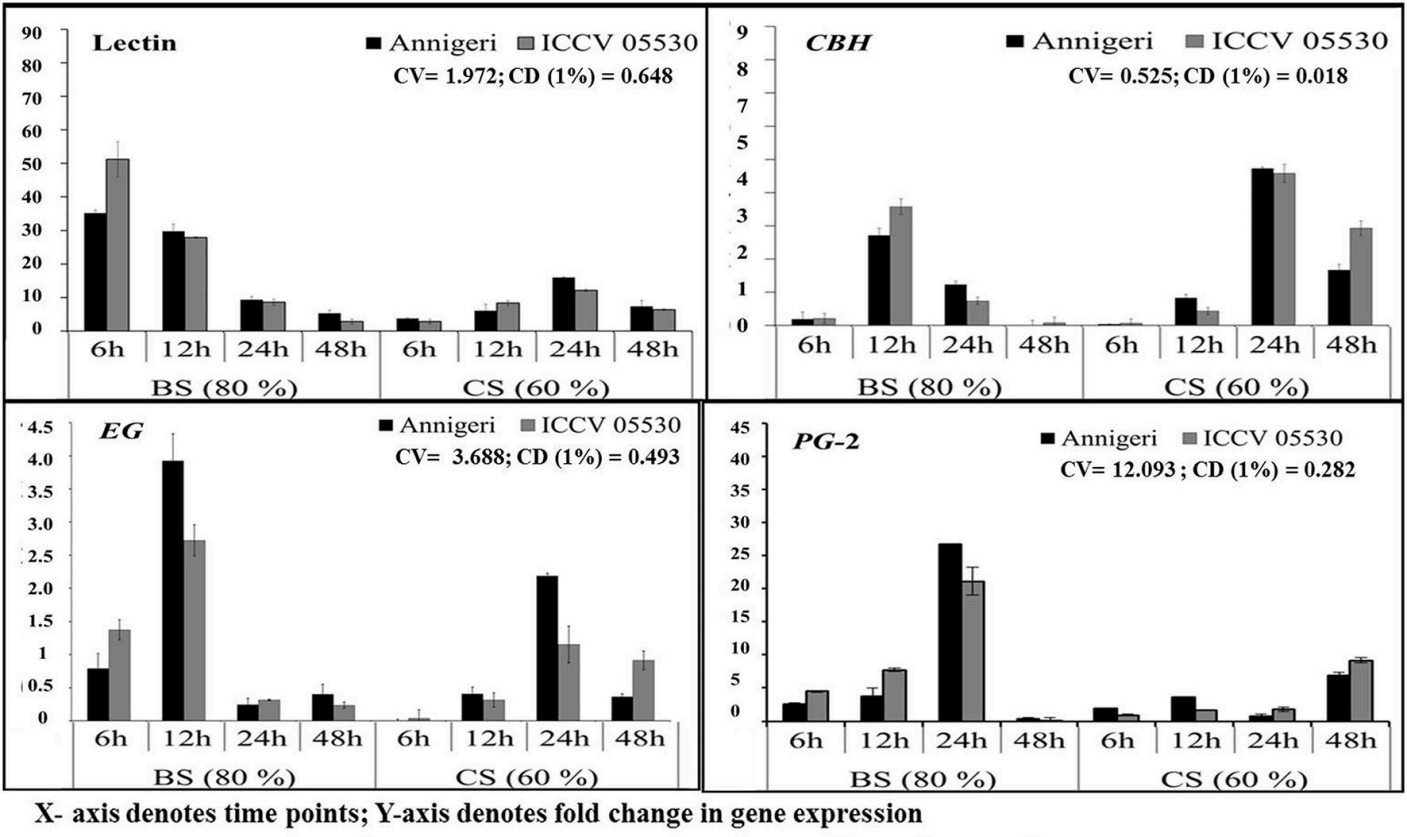
RT-qPCR analysis of different pathogenicity-causing genes of *S. rolfsii* differentially expressed at lower optimum (60%) and optimum (80%) soil moisture conditions in course of infection to chickpea. Time (in hours) and fold change in gene expression are shown on X and Y axis, respectively. Standard error of three biological replications was calculated and represented as error bar. BS and CS denote biotic stress and combined stress respectively. The statistical significance of gene expression in between the control and treated seedlings was calculated by factorial ANOVA.

The expression pattern of pathogen virulence-related genes *viz*. lectin and three genes related to plant cell wall degradation, endo β-1,4-glucanase (*EG*), cellobiohydrolase (*CBH*), and endopolygalacturinase-2 (*PG*-2) were studied in chickpea grown at optimum and lower optimum soil moisture condition. These genes were evaluated at 6, 12, 24, and 48 hpi by using real time qPCR, where only two genes (lectin and *PG*-2) were found to be highly expressed throughout the complete time-scale of infection. The trend of expression profile for the pathogenicity-causing genes was almost similar in both cultivars, Annigeri and ICCV 05530 (Figure 3). Among the four genes, lectin was highly up-regulated and showed higher transcript levels at early stages (6–12 hpi) of infection at optimum soil moisture condition. At lower optimum moisture condition, a delayed expression of lectin gene occurred which in turn was found to be much less compared to that of optimum soil moisture condition (9 and 36-fold in cvs. Annegeri and ICCV 05530, respectively). During initial stage of infection at 6 hpi, no detectable amount of fungal DNA was measured indicating insignificant colonization, but higher expression of lectin genes was recorded at the same time point, which aid in the initial attachment of the *S. rolfsii* to the plant surface. Once adhered during successful infection, the lectin gene expression was found to be downregulated at 12 hpi and further over the time period, while there was a simultaneous increase of *EG* and *CBH* gene expression at optimum soil moisture condition, where the soil moisture favored early disease establishment as conveyed by the *S. rolfsii* colonization profile (Figure 2). *EG* and *CBH* acts synergistically to breakdown the polymers of cell wall components by cleaving the internal bonds in the cellulose chain leading to growth and colonization of *S. rolfsii* (Figure S2). In lower optimum soil moisture condition, the expression of such genes was delayed owing to unfavorable soil moisture condition for *S. rolfsii* growth. The *PG*-2 gene expression was gradually up-regulated since initial time points and after successful colonization at 24 hpi, the maximum expression of *PG*-2 gene was found in optimum soil moisture condition, while the same was delayed up to 48 hpi in lower optimum soil moisture condition. The *PG*-2 gene has initially resulted in cell separation and maceration of the plant tissues which further allowed higher colonization of *S. rolfsii* in chickpea.

### Differential expression of moisture stress and biotic stress-related defense genes

To understand the possible underlying molecular mechanism during stress interaction, the expression profiling of 21 pathogen defense-related genes and five moisture-responsive genes (Table S1) in chickpea was conducted for three circumstances viz. biotic (inoculated; optimum soil moisture condition), abiotic (non-inoculated; limiting soil moisture condition) and combined pathogen and moisture stress (inoculated; lower optimum soil moisture condition) conditions (Table 1). We grouped the plant defense-related genes into three main categories which were (i) 12 pathogenesis-related (PR) genes comprising of PR-2 (β-1,3-endoglucanase), PR-4, PR-3-type chitinase (*CHI* I,*CHI* II, *CHI* III, *CHI* IV, and *CHI* V), PR-5 (thaumatin-like), PR-12 (defensin), narborin, endochitinase and germin; (ii) five phenylpropanoid pathway genes involved in phytoalexin biosynthesis comprising of phenylalanine ammonia-lyase (*PAL*-1), chalcone synthase (*CHS*), flavonoid 3'-monooxygenase (*Flav* 1), flavonoid 3' hydroxylase (*Flav* 2) and myeloblastosis family transcription factor (*MYB-Tf*); and (iii) four genes involved in reactive oxygen species (ROS) metabolism and stress-related categories comprising of lipoxygenase (*LOX*), catalase (*CAT*), peroxidase, and superoxide dismutase (*SOD*). The moisture stress responsive genes used were late embryogenesis abundant genes (*LEA-*1, *LEA-*2, and *LEA-*4), 9-cis epoxycarotenoid dioxygenase (*NCED*), and dehydration responsive element binding protein-2A (*DREB-*2A).

### Differential expression of PR genes

**Figure 4:**
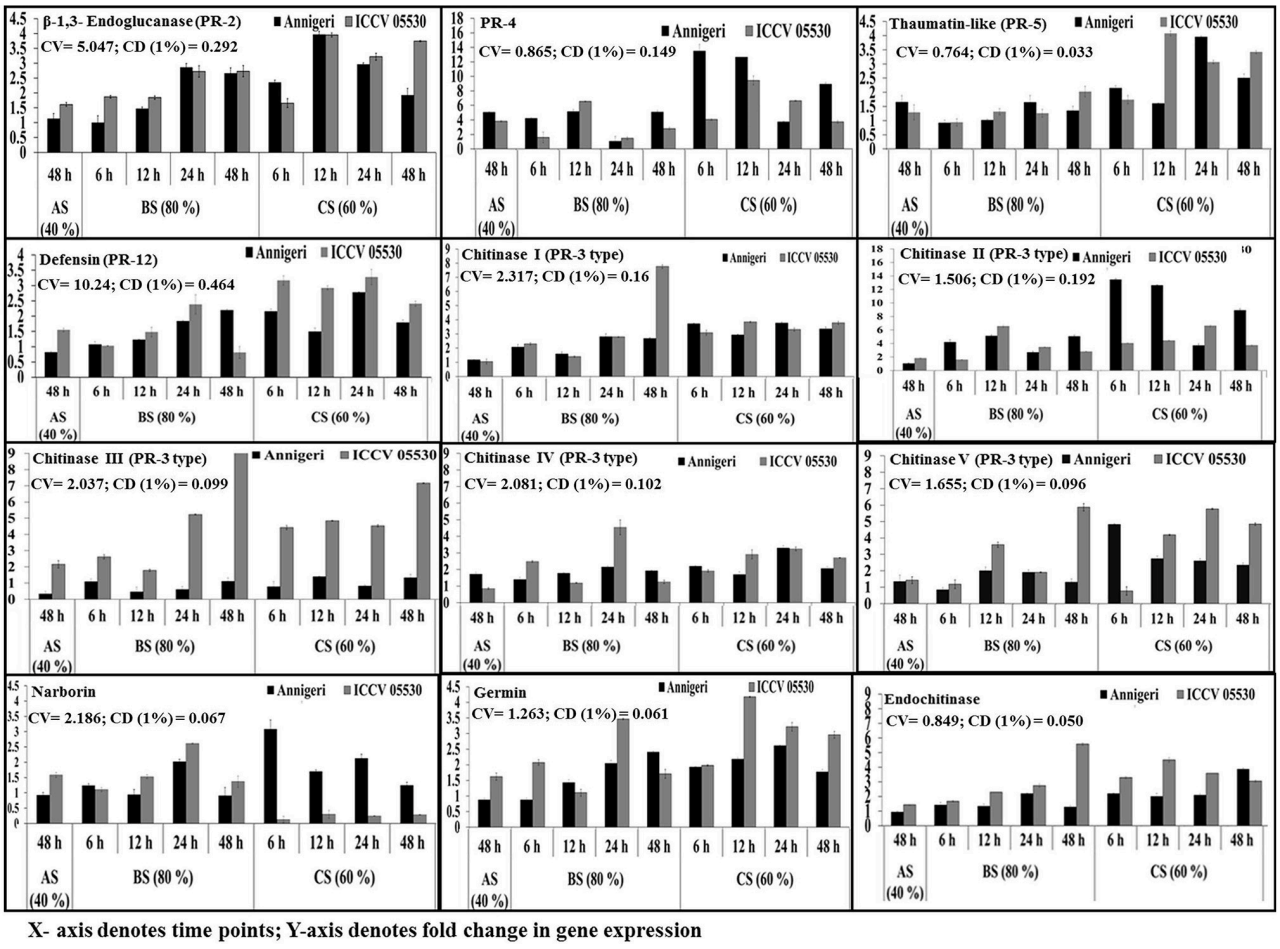
RT-qPCR analysis of different pathogenesis-related genes expressed differentially in chickpea during interaction with *S. rolfsii* at limiting (40%), lower optimum (60%) and optimum (80%) soil moisture conditions. Time (in hours) and fold change in gene expression are shown on X and Y axis, respectively. Standard error of three biological replications was calculated and represented as error bar. AS, BS, and CS denote abiotic stress, biotic stress, and combined stress respectively. The statistical significance of gene expression in between the control and treated seedlings was calculated by factorial ANOVA.

The temporal expression of PR genes showed dual (similar and differential) expression pattern within the two chickpea cultivars and also under the three different stress conditions. All PR genes except *CHI* V and narborin were found to be highly up-regulated in combined stress condition, while the expression pattern of *CHI* V gene was found to be similar in both biotic and combined stress condition. In addition, the gene for *CHI* V was specifically found to be highly expressed in cultivar ICCV 05530 at 24 hpi and 48 hpi in both combined (60% SMC) and biotic (80% SMC) stress condition. But in case of narborin gene, the cv. ICCV 05530 showed higher expression under biotic stress condition, while cv. Annigeri in combined stress. Further the genes for PR-4 and *CHI* II were over expressed in cv. Annigeri as compared to cv. ICCV 05530, while the expression pattern of *CHI* II, *CHI* IV, and PR-2 genes were found to be at par in both cultivars. The rest of the PR genes were highly expressed in cv. ICCV 05530 (Figure 4).

In abiotic stress condition (48 hpi), the *CHI* IV gene was up-regulated in Annegiri, whereas *CHI* II and *CHI* III genes were up-regulated only in ICCV 05530. On the contrary, the gene for PR-4 was expressed in both cultivars. The expression of rest of the PR genes was not found to be significant (Figure 4).

According to the time points in biotic stress condition, it was observed that at initial time point (6 hpi) there was no significant higher expression of any of the PR genes. At 12 hpi, only a single gene PR-4, was highly expressed in both cultivars. Among the expression of chitinase genes, *CHI* II was higher at 24 hpi in both cultivars. *CHI* I gene was observed to be gradually up-regulated throughout the time points in both cultivars, whereas in Annigeri higher expression of *CHI* V was found at 12 hpi. *CHI* III gene displayed a gradual increase in case of ICCV 05530 up to 24 hpi, but increase in expression was significantly higher at 48 hpi, while the expression in Annigeri remained at par in both biotic as well as combined stress throughout the time period. The genes codifying for defensin and germin showed highest expression in ICCV 05530 at 24 hpi, while the same was true for Annigeri at 48 hpi. In case of the gene for thaumatin, an opposite trend was observed to that of defensin and germin. In both cultivars the genes for narborin and glucanase exhibited highest expression at 24 hpi.

During combined stress condition at initial time point (6 hpi), *CHI* II and narborin genes showed highest expression in Annigeri, however for ICCV 05530, the highest expression was realized only at 24 hpi for the former, while the expression pattern remained at par for narborin gene throughout the time points. Also it was observed that the trend in expression pattern for PR-4 gene had gradually decreased over the time points for both cultivars. The expression pattern of genes for thaumatin, germin and endochitinase were similar, as the highest upregulation in ICCV 05530 was observed for all three at 12 hpi and the same for Annigeri at 24 hpi, except the gene for endochitinase, where the peak was observed at 48 hpi in ICCV 05530. The genes for glucanase and defensin were found to exhibit maximum expression in both ICCV 05530 and Annigeri at 12 and 24 hpi respectively. At 24 hpi, *CHI* IV gene produced a higher expression pattern in ICCV 05530 while the same for Annigeri was true at 6 hpi. A gradual increase in the expression of *CHI* III gene was observed in ICCV 05530 until the maximum was realized at 48 hpi. Only the gene for *CHI* I, produced an expression pattern that was at par throughout all the time period in both the cultivars. The only exception was at 48 hpi, where the expression of the gene was significantly high in ICCV 05530 (Figure 4).

### Differential expression of phenylpropanoid pathway genes

**Figure 5:**
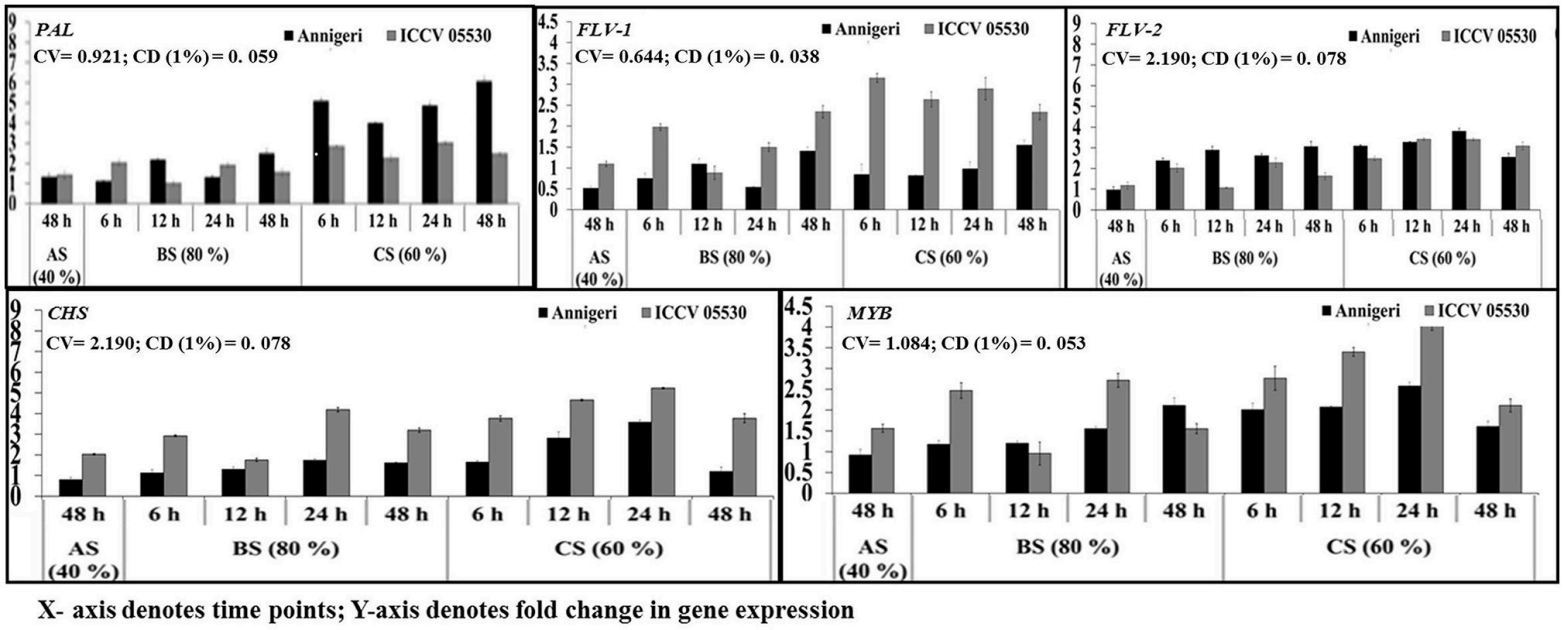
RT-qPCR analysis of differentially expressed genes involved in phenylpropanoid pathway in chickpea during interaction with *S. rolfsii* at limiting (40%), lower optimum (60%), and optimum (80%) soil moisture conditions. Time (in hours) and fold change in gene expression are shown on X and Y axis, respectively. Standard error of three biological replications was calculated and represented as error bar. AS, BS, and CS denote abiotic stress, biotic stress, and combined stress respectively. The statistical significance of gene expression in between the control and treated seedlings was calculated by factorial ANOVA.

The upregulation of genes involved in phenylpropanoid pathway was found to be high in combined stress than in biotic stress condition. During abiotic stress (48 h) *CHS* gene was up-regulated only in ICCV 05530 while expression of *PAL* 1 gene was at par in both cultivars. No significant expression was found for *MYB Tf*, *Flav* 1, and *Flav* 2 genes in both cultivars, while genes coding for MYB Tf, FLAV 2 were downregulated in abiotic stress than control in cv. Annigeri (Figure 5).

During biotic stress, very low expression of *Flav* 1 and *Flav* 2 genes were found throughout infection period in both cultivars. *PAL* 1 gene expression was observed to be at par in all the time points as well as for both cultivars. For the gene codifying for *CHS*, no significant expression was found during biotic stress except 24 hpi where the maximum upregulation of 2-fold over control was observed.

In combined stress, expression pattern of *Flav* 1 gene was similar in both cultivars and at par in all the time points of up to 4-fold than control. In Annigeri, *Flav* 2 was found to be downregulated at all the time points except 48 hpi, where as in ICCV 05530, the expression level was very low up to 2.5-fold at par throughout the time points. The maximum expression of *PAL* 1 gene of up to 6-fold was observed at 48 hpi in Annigeri while in ICCV 05530 the maximum expression was 4-fold over control. In ICCV 05530, the maximum expression of *CHS* gene of up to 5-fold was found at 12 hpi and declined during later stages. Also, in Annigeri, there was a gradual increase in the CHS gene expression of up to 4.5-fold till 24 hpi after which a decline was observed (Figure 5).

### Differential expression of ros metabolism pathway genes

**Figure 6:**
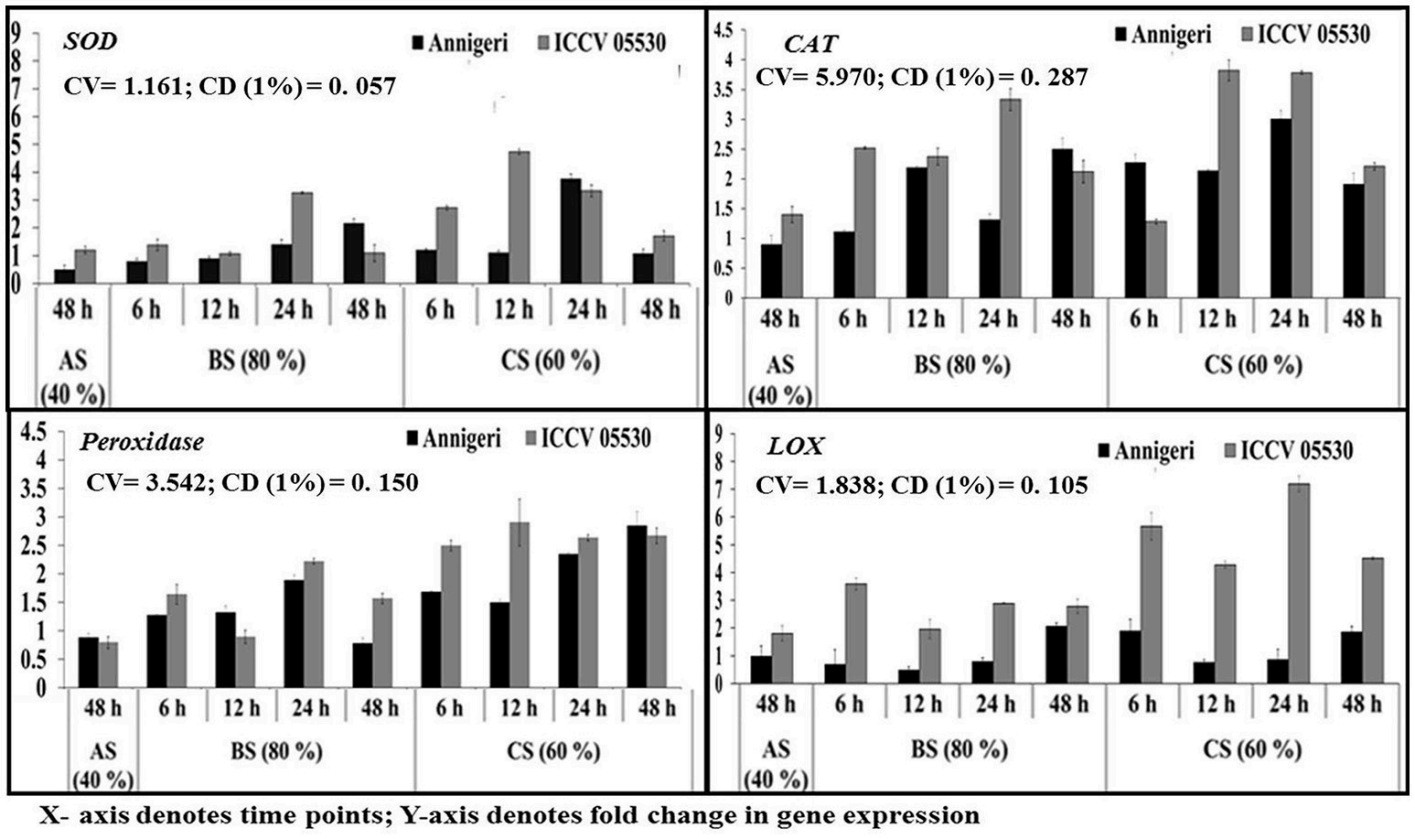
RT-qPCR analysis of differentially expressed genes involved in ROS metabolism pathway in chickpea during interaction with *S. rolfsii* at limiting (40%), lower optimum (60%), and optimum (80%) soil moisture conditions. Time (in hours) and fold change in gene expression are shown on X and Y axis, respectively. Standard error of three biological replications was calculated and represented as error bar. AS, BS, and CS denote abiotic stress, biotic stress, and combined stress respectively. The statistical significance of gene expression in between the control and treated seedlings was calculated by factorial ANOVA.

During combined stress, the expression of genes with the exception of catalase involved in ROS metabolism pathway was higher than in biotic stress condition. The expression of gene for catalase was found to be similar in both stress conditions. Apart from this, in abiotic stress (48 h) condition, only lipoxygenase gene expression was found to be significant in cv. ICCV 05530. Among the two cultivars, all the genes displayed maximum expression in ICCV 05530 than Annigeri. Among the different time points in biotic stress condition, no gene expression was observed at 6 hpi. In Annigeri, no significant expression of SOD and catalase genes was found throughout the infection period except at 12 hpi, where catalase gene was found to be expressed 2-folds than control. In ICCV 05530, maximum expression of catalase and SOD genes was found at 12 and 24 hpi respectively. In combined stress condition, maximum expression of catalase gene was found in both cultivars, where the gene coding for lipoxygenase expressed maximum only in ICCV 05530 at 48 hpi. The maximum upregulation of SOD gene was found at 12 hpi after which a decline was observed (Figure 6).

### Differential expression of moisture stress responsive genes

**Figure 7:**
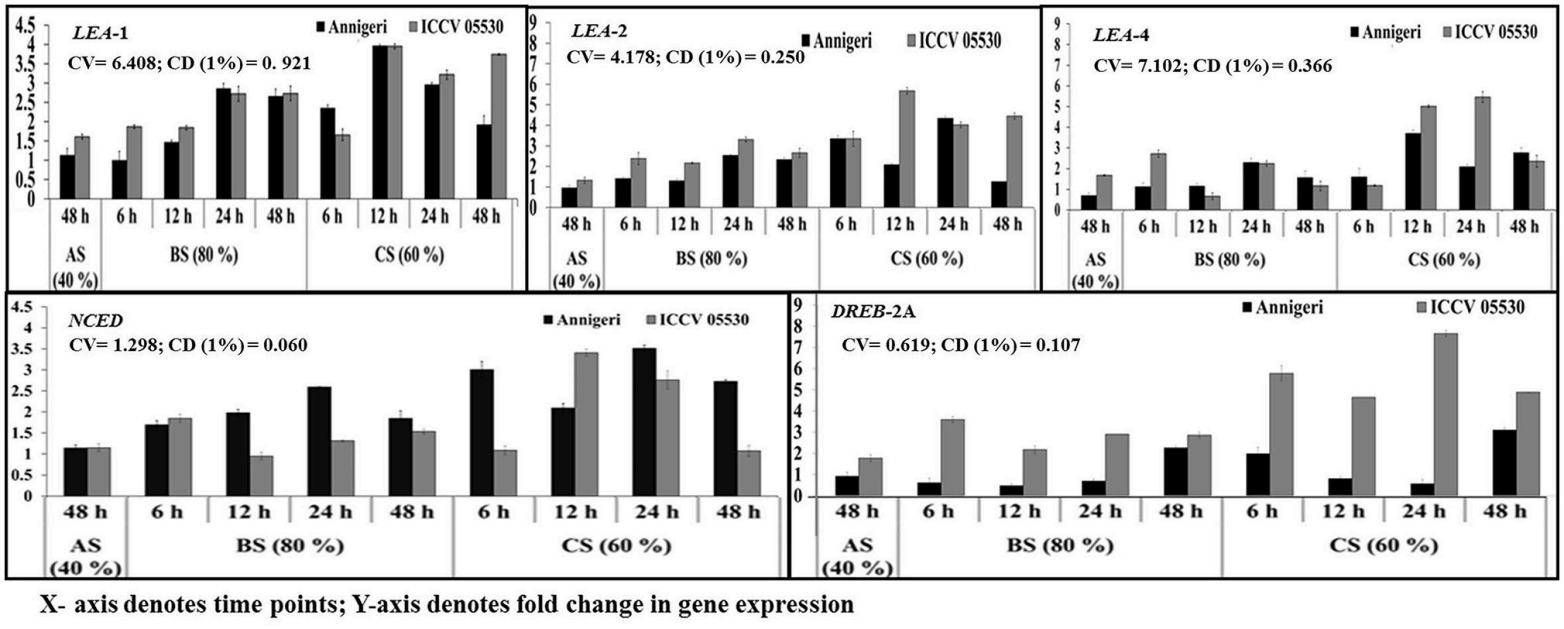
RT-qPCR analysis of different moisture stress responsive genes differentially expressed in chickpea during interaction with *S. rolfsii* at limiting (40%), lower optimum (60%), and optimum (80%) soil moisture conditions. Time (in hours) and fold change in gene expression are shown on X and Y axis, respectively. Standard error of three biological replications was calculated and represented as error bar. AS, BS, and CS denote abiotic stress, biotic stress, and combined stress respectively. The statistical significance of gene expression in between the control and treated seedlings was calculated by factorial ANOVA.

According to the profile, the cv. ICCV 05530 resulted in more expression of such genes except *NCED* in all three circumstances than the cv. Annigeri. Among the different stress conditions studied, for both cultivars, expression of those genes at combined stress was found to be more than that of the individual stress. Within the *LEA* genes, expression of *LEA* 1 was high as compared to *LEA* 2 and *LEA* 4 in both cultivars under all three stresses. In ICCV 05530, the maximum expression of *LEA* 1 gene of up to 56-fold was detected at 24 hpi, while in Annigeri the same of up to 51-fold was detected at 48 h. In both the cultivars there was a gradual increase in the expression of *NCED* and *DREB* 2A genes for up to 24 hpi, after which a decline was observed (Figure 7).

## Discussion

Being one of the most extensively fluctuating and acutely affecting climatic variables among other environmental factors, soil moisture has a profound influence over the soil pH, aeration and availability of nutrients, thereby affecting the growth and population dynamics of the soil microorganisms such as bacteria, oomycetes, fungi etc. (Rousk and Bååth, 2011). Despite its importance, moisture relations of soil microorganisms are a less ventured aspect and even more in phytopathogenic fungi. The existing data on disease severity and incidence caused by soil fungi in relation to soil moisture content are often time consuming. In the case of emerging collar rot disease of chickpea, high disease incidence in field level is often reported to be associated with high soil moisture condition (Sharma and Ghosh, 2017). Currently, there is limited information about molecular basis of chickpea and *S. rolfsii* interaction with soil moisture. Hence in this study, we reviewed molecular response of both chickpea and *S. rolfsii* during collar rot development under different soil moisture conditions.

In our *in vitro* study with collar rot in chickpea, a higher percentage of collar rot incidence in chickpea was observed at high soil moisture condition (80–100% SMC). The development of collar rot in the field was correlated with growth and colonization. *S. rolfsii* colonization was found to be maximum toward higher SMC (80 and 100% SMC) whereas, the pathogen growth was found to decline in lower SMC (60%) and the least growth was found in limiting SMC (40%). Prasad and Saifulla (2012) observed a decrease in growth and population of *F. udum* causing pigeonpea wilt at 25 to 50% SMC while 75% SMC was favorable for the same.

During infection, phytopathogenic fungi produce lectins and an array of digestive enzymes to degrade the plant cell wall and establish pathogenicity (Sharma et al., 2016). Lectins are carbohydrate binding proteins highly specific for sugar moieties and helps pathogen to bind the host surface (Rutishauser and Sachs, 1975). The enzymes EG, CBH and PG-2 work on different polysaccharides like cellulose, hemicellulose and pectin to loosen the cell wall integrity in plants and help the fungi for nutrient acquisition. EG is an enzyme that catalyzes the hydrolysis reaction to breakdown the glucosidic bonds in polysaccharide, e.g., glucan present in plant cell wall. Post-catalytic reaction of EG on cellulose, the enzyme CBH catalyzes it into cellobiose and glucose through breakdown the 1,4-β-D-glycosidic linkages (Polizeli et al., 2016), and the enzyme endopolygalacturinase-2 hydrolyzes the α-1,4-glycosidic bonds between galacturonic acid residues, a significant component of the pectin network comprising plant cell wall (Figure S2).

In our study, at the time of infection, lectin gene showed high level of expression at 6 hpi in optimum SMC, indicating the early adhesion of *S. rolfsii* in chickpea at initial stage of collar rot development. In lower optimum SMC, delayed expression of lectin gene, resulted in the infection to be adjourned up to 24 hpi. After attachment of *S. rolfsii* on host plant, at 12 hpi, the *EG* and *CBH* genes expressed synergistically which led to breakdown of cellulose present in cell wall by serial cleaving of chemical bonds which produced simple organic sugars and it acquires in the form of nutrients by *S. rolfsii* and helps it to successful colonization within the host tissue. The gene for PG-2 expressed in early stage of infection in optimum SMC, and gradually increased with the disease progression, in accordance with the results reported previously in other plant-fungal interactions (Shieh et al., 1997). On the contrary, in lower optimum SMC, the expression of *PG*-2 gene was detected only at the later stages (24 and 48 hpi). These expressions in late stages suggests attempt of the pathogen to acquire nutrients in growth limiting environment. The expression of PGs has earlier been reported under nutrient-deprived conditions and also in the presence of pectin (Yao et al., 1999). The expression patterns of the candidate pathogenicity-causing genes analyzed in this study correlate well with colonization pattern of *S. rolfsii* in chickpea plants.

Plants have their own defense mechanisms to defend against multiple stresses (biotic and abiotic) and they may modify it according to occurrence of stress present in its immediate surroundings (Mickelbart et al., 2015; Padaria et al., 2015). In a condition when the multiple stresses occur at once in plants, the net impact of each stress during their interaction and the corresponding responses to combined stress is quite different when compared to individual stresses alone (Choi et al., 2013; Ramegowda et al., 2013; Ramegowda and Senthil-Kumar, 2015). In present study, we have compared the molecular response of several defense-related as well as moisture stress responsive genes in chickpea during combined stress of *S. rolfsii* and soil moisture. The present study reports the expression profiling of defense-related transcripts during *S. rolfsii* interactions with chickpea host system, along with the comparison of transcriptional response during soil moisture stress or/and dual stress of soil moisture and pathogen. Jogi et al. (2016) also conducted similar research in the identification of differentially expressed genes during early interactions between the stem rot causing *S. rolfsii* and peanut (*Arachis hypogea*) cultivars with increasing disease resistance levels.

Principal observations from this study is on expression profiling of defense-related transcripts during *S. rolfsii* interactions with the host system, along with the comparison of transcriptional response during soil moisture stress alone or dual stress with pathogen. During collar rot development in chickpea plants at optimum SMC, the usual expression of the most defense response genes (PR-2, PR-5, PR-12, *CHI* I, *CHI* III, narborin, defensin, germin, and endochitinase) was not significant at early stages of infection. Moreover, their expression was up-regulated more during combined stress condition involving soil moisture (60% SMC) and pathogen, thereby delaying the collar rot development. Even at 48 hpi, there was negligible expression of *CHI* II and *CHI* IV genes apart from the above genes in chickpea plants maintained at limiting (40%) SMC. The reason might be because 48 h duration of stress to chickpea plants at limiting SMC was not sufficient for induction of genes related to low soil moisture stress. At optimum (80 %) SMC some of the PR-3 type genes (*CHI* I, *CHI* III and *CHI* V) and gene for endochitinase had significantly over expressed up to 48 hpi in ICCV 05530 as compared to Annigeri, supporting initial resistance in the cultivar. High constitutive levels of chitinases, PR-2 and defensin in chickpea during combined stress was observed which may signify its role in releasing fungal cell wall elicitors at the onset of infection Previous reports showed that degradation products of fungal chitin and glucan functions as elicitors and trigger the hypersensitive response (HR) in host plants (Jones and Dangl, 2006). In earlier studies, Gupta et al. (2016) also observed similar findings in accordance to our results in *Arabidopsis thaliana* during soil moisture and pathogen interaction, and Boominathan et al. (2004) with chickpea and soil moisture interaction. Chitinases from plants can inhibit fungal growth by degrading chitin present in the fungal cell walls; also, the resultant chitinolytic breakdown products are able to further elicit other defense reactions within the plants (Figure S2). Antifungal properties of defensins induced its expression upon fungal attack and have been reported (Penninckx et al., 1996). Germin like proteins possessing strong oxalate oxidase activity has reported to be involved in several biotic and abiotic stress-related processes (Woo et al., 2000). Increased expression of endochitinase during biotic stress and combined stress indicates involvement of ethylene signaling pathway. Over-expression of endochitinase gene in *Nicotiana tabacum* and *Brassica napus* has reduced *R. solani* symptoms (Broglie et al., 1991). Induction of pathogen defense responsive genes under varied drought stress and vice-versa has been noted which is well in concurrence with the previous reports Liu et al., 2013; Ramegowda et al., 2013.

Phenylalanine ammonia-lyase (PAL) has an important role in plant systemic resistance through its biosynthesis of salicylic acid and active involvement in phenylpropanoid metabolic network (Chaman et al., 2003). Different classes of phenylproponoids are synthesized and accumulated in response to pathogen infection in leguminous plants (Gurjar et al., 2012). In flavonoid biosynthetic pathway, CHS plays an important catalytic role during the initial stage (Figure S2). *CHS* gene expression is reported to be induced in plants under various biotic and abiotic stress conditions. CHS expression results in accumulation of several flavonoid compounds, thereby inducing the salicylic acid defense pathway (Dao et al., 2011). In our study, the significant expression of the four key enzymes (phenylalanine ammonia-lyase, chalcone synthase, flavonoid 3'-monooxygenase and flavonoid 3' hydroxylase) in chickpea plants with combined stress positively correlates with earlier reports.

ROS accumulation is associated with plant defense against pathogens (Hückelhoven and Kogel, 2003). However, ROS resulting from biotic and abiotic stresses can also result in cause severe cellular damage and is therefore tightly regulated and detoxified by complex enzymatic and non-enzymatic mechanisms (Figure S2). Antioxidant enzymes such as POD, SOD, and CAT are activated to scavenge the redundant ROS and play a crucial role in the antioxidant systems to protect plant cells from damage (Naya et al., 2007). In our study, a pronounced increase in the POD and SOD activities were observed in collar rot infected chickpea plants, especially, the plants maintained at lower optimum SMC. The results indicated that chickpea plants at lower optimum SMC had gained, to some extent, more ability to withstand against disease than the plants maintained at optimum SMC.

Gene encoding a chaperon protein like LEA, involved in preventing water stress as well as inducing an aggregation of sensitive proteins (Olvera-Carrillo et al., 2010) is also expressed during biotic and combined stress apart from soil moisture stress (Figure S2). Another soil moisture stress specific *NCED* 3, an abscisic acid (ABA) biosynthesis gene have been shown to be up-regulated during soil moisture and combined stress leading to accumulation of ABA (Iuchi et al., 2001; Padaria et al., 2016). DREBs are an important group of plant transcription factors responsible for regulating the expression of many stress-inducible genes generally in an ABA-independent manner. They also play a critical role in improving the abiotic stress tolerance ability of plants Lata and Prasad, 2011). Similarly, the significantly higher expression of few soil moisture stress inductive genes (*LEA* family, *NCED* 3, and *DREB*) was observed in collar rot infected chickpea plants maintained at lower optimum SMC compared optimum SMC.

Our study indicates that the combined stress imposition initiated a much stronger and earlier response than when only pathogen inoculation or moisture stresses were applied individually. Soil moisture stress alone incited a slower response compared to pathogen inoculation and the combined stresses. Although differences in the transcript expression were noted following the individual pathogen and soil moisture stress as well as, in combined pathogen and soil moisture stress, there was a substantial degree of overlap recorded. Many genes reported to be pathogen-induced were also influenced by the soil moisture stress, although the response was lower compared to inoculation of pathogen alone. These findings provide evidence of induction in basal defenses as a contributory factor for the enhanced resistance response.

## Conclusion

The present study demonstrates that, soil moisture stress (limiting and upper optimum) reduces the multiplication of *S. rolfsii in planta*. Delayed and significantly lower expression of pathogenicity-causing genes of *S. rolfsii* was observed in the infected chickpea plants exposed to lower SMC irrespective of the cultivars. Inversely, more expression of defense-related genes at lower SMC had additional effects on inhibition of fungal growth and in decisive decrease of collar rot severity. Therefore, the net effect of soil moisture stress on gene expression in both systems *viz*. host and pathogen could lead to adjourned disease establishment and symptom development. The true explanation of these findings could be correlated with collar rot disease occurrence in chickpea seedlings at optimum and upper optimum soil moisture conditions, where few defense related genes like chitinase and endochitinase were over-expressed. The functional validation of these genes will assist in further understanding the chickpea defense system against *S. rolfsii*.

## Author contributions

AT and TR in consultation with MS conceived, designed and initiated the study. AT and TR contributed equally and were responsible for analysing and interpretation of results and initial drafting of the manuscript. UC helped in setting up of experiments. TR conducted validation studies, RG, and AT contributed in further improvement of analysis and provided inputs in drafting the manuscript. DC helped in analysis and manuscript writing. MS provided critical inputs at various stages of the study and edited the manuscript. All authors read and approved the manuscript.

### Conflict of interest statement

The authors declare that the research was conducted in the absence of any commercial or financial relationships that could be construed as a potential conflict of interest.

## Supplementary material

Figure S1 Calibration of qPCR for quantification of *S. rolfsii* Standard curve showing the correlation between the log10 DNA quantity (ng) vs. the Ct values for 10-fold dilution of *S rolfsii* pure genomic DNA. The single peak of targeted amplicon at melting temperature (Tm) 81.5°C indicates the specificity of the qPCR primers to *S. rolfsii*. No contaminating product was detected in PCR reaction.

Figure S2 Schematic diagram of molecular interaction between chickpea and *S. rolfsii* under low soil moisture condition. (Courtesy of diagram of plant cell wall: www.thoughtco.com/cell-wall-373613).

Table S1 Primer sequences, correspondence sequence ID used for real-time PCR and gene expression profile in chickpea.

Table S2 Collar rot disease incidence in chickpea cultivars.
