# Supplementary figures and images for: Exploring Combined Effect of Abiotic (Soil Moisture) and Biotic (Sclerotium rolfsii Sacc.) Stress on Collar Rot Development in Chickpea
Source: Front Plant Sci. 2018 Aug 15;9:1154. doi: 10.3389/fpls.2018.01154 (PMC6104659; doi:10.3389/fpls.2018.01154)

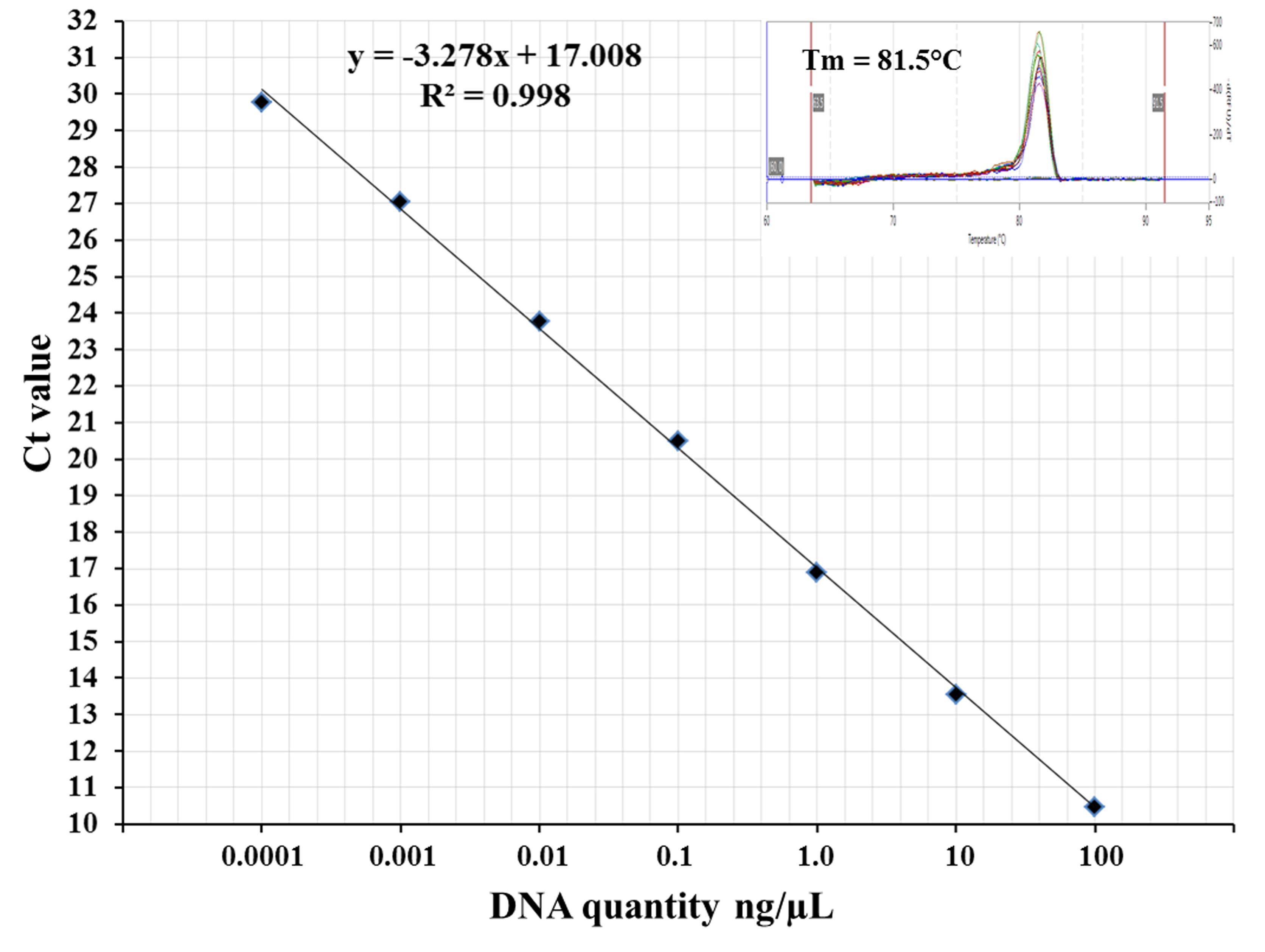

Supplement: Figure S1 — Calibration of qPCR for quantification of S. rolfsii Standard curve showing the correlation between the log10 DNA quantity (ng) vs. the Ct values for 10-fold dilution of S rolfsii pure genomic DNA. The single peak of targeted amplicon at melting temperature (Tm) 81.5°C indicates the specificity of the qPCR primers to S. rolfsii. No contaminating product was detected in PCR reaction. [file Image_1.JPEG]

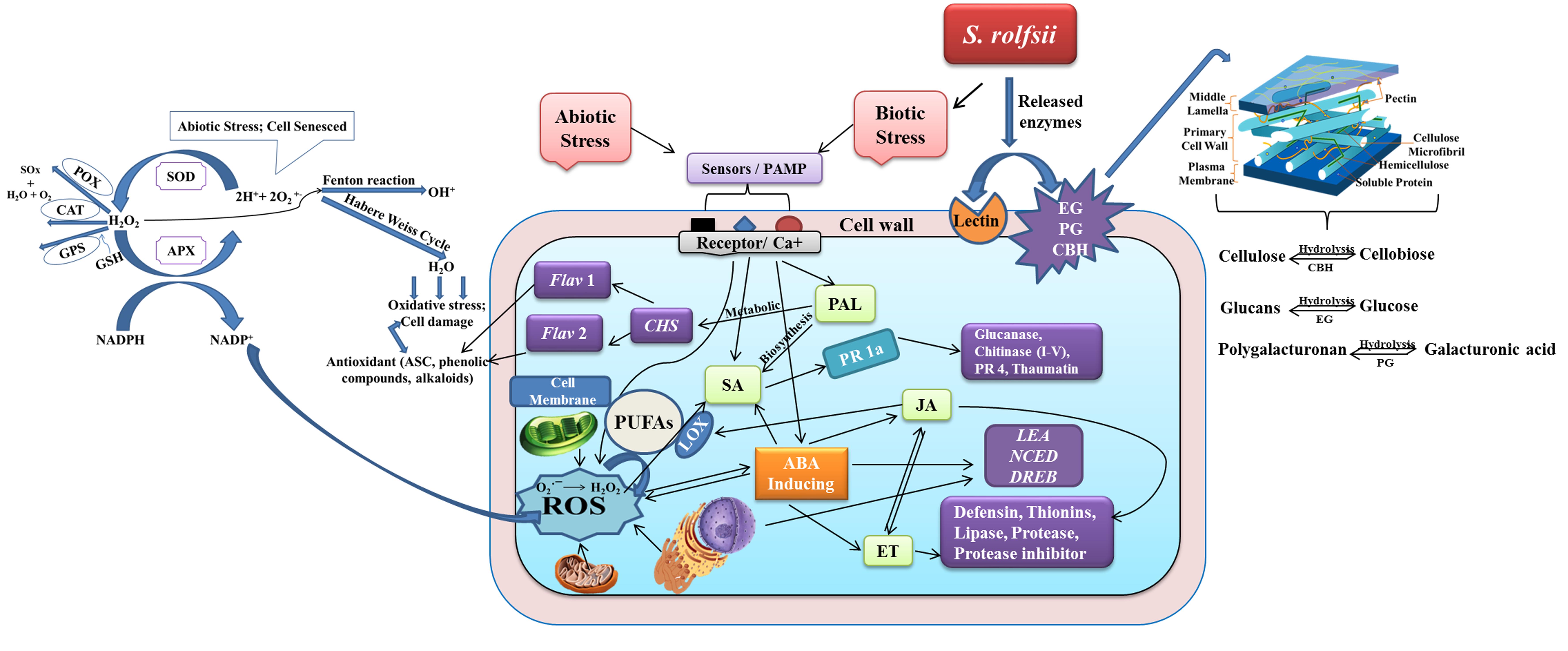

Supplement: Figure S2 — Schematic diagram of molecular interaction between chickpea and S. rolfsii under low soil moisture condition. (Courtesy of diagram of plant cell wall: www.thoughtco.com/cell-wall-373613). [file Image_2.JPEG]
