# Supplementary material for: Exploring Combined Effect of Abiotic (Soil Moisture) and Biotic (Sclerotium rolfsii Sacc.) Stress on Collar Rot Development in Chickpea
Source: Front Plant Sci. 2018 Aug 15;9:1154. doi: 10.3389/fpls.2018.01154 (PMC6104659; doi:10.3389/fpls.2018.01154)
Supplement: Table S1 — Primer sequences, correspondence sequence ID used for real-time PCR and gene expression profile in chickpea. [file Table_1.docx]

**TABLE S1**│**Primer sequences, correspondence sequence ID used for real-time PCR and gene expression profile in chickpea.**

| **Genes coding for** | **Transcript ID*** | **Primers name** | **Primer sequence (5’→3’)** | **Amplicon size** |
| --- | --- | --- | --- | --- |
| ***Sclerotium rolfsii* pathogenicity-causing genes** | | | | |
| Lectin | JN811676.1 | qSR_lectin(F)  qSR_lectin(R) | ATCTACCAGACCAACCCGAA  CCATTGTGAGGACATGCTGAT | 112 |
| Cellobiohydrolase (*CBH*) | AB103461.1 | qSR_CBH(F)  qSR_CBH(R) | CACCCTTCTCTCACTGTTTCTC  CCGTGTAGCAGTTGGTGTAA | 127 |
| Endo β-1,4-glucanase (*EG*) | E37750.1 | qSR_EG(F)  qSR_EG(R) | GCCATATCTCGGTGGTGTAAA  AGCGAAAGGAATACGGAAGAG | 148 |
| Endopolygalacturinase-2 (*PG*-2) | AB491192.1 | qSR-PG2(F)  qSR-PG2(R) | GTCAGCGGCATCACTATCAA  GCGATGGAACCAATGGAGATA | 206 |
| ***Sclerotium rolfsii* reference gene** | | | | |
| 5.8S ribosomal RNA gene | gi\|658115857 | qSR-5.8S(F)  qSR-5.8S(R) | AACGGATCTCTTGGCTCTTG  CACATCCAAGCCTTGACAAATAC | 104 |
| **Chickpea pathogenesis-related (PR) genes** | | | | |
| β-1,3-endoglucanase (PR-2) | TC11637 | qCP_βgluc(F)  qCP_βgluc(R) | GGTCGGCTACTTCGTATGATAAC  TCCTTCTTTCTCCACCAAATCC | 216 |
| PR-4 | TC24032 | qCP_PR4(F)  qCP_PR4(R) | GAGGACTCCAAACGTGAGATTG  GGCCACTGTTTCAGGAGAAT | 135 |
| Thaumatin-like (PR-5) | AJ010501.1 | qCP_thaumatin(F)  qCP_thaumatin(R) | TCAGTTGCACAGCCGATATT  GTGCTAGTTGGGTCATCTTGAG | 205 |
| Defensin (PR-12) | gi\|84569908 | qCP_Defensin(F)  qCP_Defensin(R) | TGGCTTGTGCTTCCTCTT  GTGCACCAACAACGAAAGTC | 192 |
| PR-3-type chitinase I (*CHI* I) | TC11756 | qCP_Chitinase I(F)  qCP_Chitinase I(R) | TTGCTACCGACCCTGTTATTT  ATCCGATCTTGAACTCTGCTATC | 220 |
| PR-3-type chitinase II (*CHI* II) | TC18011 | qCP_Chitinase II(F)  qCP_Chitinase II(R) | CTGCACCTGATGGACCATATT  GGACACAATCTCAGGGTTCTT | 215 |
| PR-3-type chitinase III (*CHI* III) | TC10517 | qCP_Chitinase III(F)  qCP_Chitinase III(R) | CTTGCAACACAAACAACTACCA  TCAGCGGAGTTCAGAGAGTA | 217 |
| PR-3-type chitinase IV (*CHI* IV) | TC20957 | qCP_Chitinase IV(F)  qCP_Chitinase IV(R) | ATGCACCTAGTTCTACTACCAATG  ACTTACGTCCAGTTTCTTGTGT | 209 |
| PR-3-type chitinase V (*CHI* V) | TC28250 | qCP_Chitinase V(F)  qCP_Chitinase V(R) | GTAATGGGCCGAGATCATATCC  GGTCCTTTAACTCCCATGTTCT | 243 |
| Narborin | TC22821 | qCP_Narborin(F)  qCP_Narborin(R) | GCAGCAATACAACGAAGTCAAG  GTAGAACTGATAGTCCACCCAATC | 105 |
| Germin | TC25462 | qGermin(F)  qGermin(R) | GGGTCTAAATCCTCCACATACTC  GAGACCAATTGGGAACACAAAC | 157 |
| Endochitinase | TC10517 | qEndoChi(F)  qEndoChi(R) | GTCCTTACCCTGATGCTCATTT  GTCCATTGATTCCAAGCATTAACA | 142 |
| **Chickpea defence-related phenylpropanoid pathway genes** | | | | |
| Phenylalanine ammonia-lyase (*PAL*) | TC00723 | qCP_PAL1(F)  qCP_PAL1(R) | ACTCTTCCCGATCCACTCA  CTCGACACGAACACCACTATC | 180 |
| Flavonoid 3'-monooxygenase (*Flav*-1) | TC06119 | qCP_Flav1(F)  qCP_Flav1(R) | CAATGGACACTTCTGCAACATC  GCCACAGGATGGAGTCTAAAG | 193 |
| Flavonoid 3' hydroxylase (*Flav*-2) | TC07559 | qCP_Flav2(F)  qCP_Flav2(R) | TTCAGGGTCGAAAGCAGTAAA  CCATAAGCTCAACCACCATAGA | 98 |
| Chalcone synthase (*CHS*) | TC05545 | qCP_CHS(F)  qCP_CHS(R) | GAATACATGGCACCTTCATTGG  AGGCATGTCAACACCACTT | 162 |
| Myeloblastosis family transcription factor (*MYB-Tf*) | TC07685 | qCP_MYB(F)  qCP_MYB(R) | GGACTGCTGAGGAAGACAAA  AGACGAGCATGGAGATCAATAAC | 205 |
| **Chickpea defence-related ROS metabolism pathway genes** | | | | |
| Superoxide dismutase (*SOD*) |  | qCP_SOD(F)  qCP_SOD(R) | ACATTTGCTACCTCTCCCTCACCT  TCGGGTAAGACATCGTCGGTATGT | 172 |
| Catalase (*CAT*) |  | qCP_Cat(F)  qCP_Cat(R) | GGCGGTACGTTTACGATTTACGCT  ACCTATCACGGGTCAGCACGATTT | 191 |
| Peroxidase | gi\|828330586 | qCP_Perox(F)  qCP_Perox(R) | GTTCAGGGTTGTGATGGTTCTA  TAACATCACGGGTTGCCATAG | 199 |
| Lipoxygenase (*LOX*) | AJ276265.1 | qCP_LOX(F)  qCP_LOX(R) | TTAAGACATGGGTCCAAGAGTATG  GAGCAGAAGCAGTCCATATGAT | 204 |
| **Chickpea defence-related drought responsive genes** | | | | |
| Late embryogenesis abundant-1 (*LEA*-1) | AJ224518.1 | qCP_ LEA1(F)  qCP_ LEA1(R) | GTGAGACCATGGGCCGAAC  TTGGGCTGTCTGACTGGT | 199 |
| Late embryogenesis abundant-2 (*LEA*-2) | AJ224519.1 | qCP_ LEA2(F)  qCP_ LEA2(R) | AGGTGCAACTGATGCTGTGA  GCGTTGAATAAAAACCAAATTACGA | 180 |
| Late embryogenesis abundant-4 (*LEA*-4) | GU247512.1 | qCP_ LEA4(F)  qCP_ LEA4(R) | GCTCACACCCGATTGGAACT  AGACTAAACTTTGTGCAGTCCTT | 200 |
| 9-cis epoxycarotenoid dioxygenase (*NCED*) | AB771415.1 | qCP_ NCED(F)  qCP_ NCED(R) | ACCCACGTGTCCAAATCTCC  CGGCTACCGGTTCGTAATGT | 160 |
| Dehydration responsive element binding protein-2A (*DREB*-2A) | DQ321719.1 | qCP_ DREB(F)  qCP_ DREB(R) | AGCACATGTTAGTGAAAAGCCA  CAAGGCGGGCGTTCAGTT | 176 |
| **Chickpea reference gene** | | | | |
| Actin | AJ012685 | qCP_Actin(F)  qCP_Actin(R) | GTGGTGGTTCTACTATGTTCCC  CTGTATTTCCTCTCTGGTGGTG | 115 |
