# Supplementary material for: Exploring Combined Effect of Abiotic (Soil Moisture) and Biotic (Sclerotium rolfsii Sacc.) Stress on Collar Rot Development in Chickpea
Source: Front Plant Sci. 2018 Aug 15;9:1154. doi: 10.3389/fpls.2018.01154 (PMC6104659; doi:10.3389/fpls.2018.01154)
Supplement: Table S2 — Collar rot disease incidence in chickpea cultivars. [file Table_2.docx]

**TABLE S2 | Collar rot disease incidence in chickpea cultivars.**

| **Cultivar** | **Soil moisture condition (%)** | **Hour post inoculation (hpi)** | | |
| --- | --- | --- | --- | --- |
|  |  | **48 hpi** | **96 hpi** | **144 hpi** |
| Annigeri | 40 | 0 | 0 | 0 |
|  | 60 | 33.3 | 57.1 | 61.9 |
|  | 80 | 76.2 | 90.5 | 95.2 |
|  | 100 | 71.4 | 85.7 | 90.5 |
| ICCV 05530 | 40 | 0 | 0 | 0 |
|  | 60 | 0 | 23.8 | 57.1 |
|  | 80 | 9.5 | 28.6 | 90.5 |
|  | 100 | 4.8 | 28.6 | 85.7 |

The statistical analysis was done by three-way factorial ANOVA with the 5% level of significance (*p* < 0.05).
